# Age-Differential Effects of Job Characteristics on Job Attraction: A Policy-Capturing Study

**DOI:** 10.3389/fpsyg.2017.01124

**Published:** 2017-06-30

**Authors:** Hannes Zacher, Bodil T. Dirkers, Sabine Korek, Brenda Hughes

**Affiliations:** ^1^Institute of Psychology, Leipzig UniversityLeipzig, Germany; ^2^School of Management, Queensland University of TechnologyBrisbane, QLD, Australia; ^3^Department of Psychology, University of GroningenGroningen, Netherlands

**Keywords:** age, job design, job characteristics, job attraction, policy-capturing

## Abstract

Based on an integration of job design and lifespan developmental theories, Truxillo et al. (2012) proposed that job characteristics interact with employee age in predicting important work outcomes. Using an experimental policy-capturing design, we investigated age-differential effects of four core job characteristics (i.e., job autonomy, task variety, task significance, and feedback from the job) on job attraction (i.e., individuals' rating of job attractiveness). Eighty-two employees between 19 and 65 years (*M*_age_ = 41, *SD* = 14) indicated their job attraction for each of 40 hypothetical job descriptions in which the four job characteristics were systematically manipulated (in total, participants provided 3,280 ratings). Results of multilevel analyses showed that the positive effects of task variety, task significance, and feedback from the job were stronger for younger compared to older employees, whereas we did not find significant age-differential effects of job autonomy on job attraction. These findings are only partially consistent with propositions of Truxillo et al.'s (2012) lifespan perspective on job design.

## Introduction

Populations and workforces around the globe are aging and becoming increasingly age diverse (Hedge and Borman, 2012; Truxillo et al., 2015). This implies that organizations have to identify effective ways to attract highly qualified younger and older job applicants. So far, however, only a small number of survey studies have examined age-differential associations between job characteristics and work outcomes (e.g., Zaniboni et al., 2013, 2014). Using an experimental policy-capturing design (Aguinis and Bradley, 2014), the goal of the present study was to investigate which jobs are most attractive to younger and older workers, respectively. Adopting a lifespan perspective on job design, Truxillo et al. (2012) suggested that young and older workers have different preferences with regard to job characteristics. They offered a model, based on an integration of job design and lifespan developmental theories, that outlines possible moderating effects of age on relationships between various job characteristics and work outcomes.

With this article, we aim to contribute to the literature on age and job design in three ways: First, using an experimental vignette methodology design, we conduct a rigorous empirical investigation of core propositions of Truxillo et al.'s (2012) lifespan perspective on job design. In particular, we investigated how four core job characteristics influence workers' job attraction, and whether these influences vary depending on age (see also Griffiths, 1999; Truxillo and Zaniboni, 2017).

Second, Truxillo et al. (2012) included job satisfaction, work engagement, and performance as outcome variables in their model. We extend research on this model by focusing on job attraction, or individuals' ratings of job attractiveness, as an outcome variable. Job attraction describes the extent to which applicants would like to carry out a given job (Singh, 1975; Rynes and Lawler, 1983; Rynes and Miller, 1983). In the context of an aging workforce and a “war for talent,” it is important to investigate predictors of job attraction, as organizations are interested in attracting and hiring highly qualified or qualifiable younger and older job applicants (Zacher et al., in press).

Job attraction is a popular criterion in the recruitment literature. It differs from other recruitment-related constructs such as job pursuit intentions, acceptance intentions, and job choice (Chapman et al., 2005). Job attraction is typically measured by asking applicants to provide an overall evaluation of the attractiveness of the job they are applying for (e.g., “How attractive is the job to you?”; Saks et al., 1994). In contrast, job pursuit intentions include “a person's desire to submit an application, attend a site visit or second interview, or otherwise indicate a willingness to enter or stay in the applicant pool without committing to a job choice” (Chapman et al., 2005, p. 929). Acceptance intentions describe “the likelihood that an applicant would accept a job offer if one were forthcoming” (Chapman et al., 2005, p. 929). Finally, job choice is an action that entails “choosing whether to accept a real job offer involving an actual job” (Chapman et al., 2005, p. 929).

According to Chapman et al. (2005), the influence of job characteristics on job attraction can be explained by objective factor theory (Behling et al., 1968), which states that applicants form their job-related attitudes based on evaluations of objective job or position characteristics. Surprisingly, however, research on the effects of motivational job characteristics, particularly those proposed by the job characteristics model (i.e., job autonomy, task variety, task identity, task significance, feedback from the job; Hackman and Oldham, 1976), is very sparse. We identified only one early study by Farh and Scott (1983), which showed that three of these job characteristics (i.e., job autonomy, task variety, and feedback from the job) are positively and moderately related to job attraction. Thus, our study also contributes to the literature by investigating general effects of job characteristics on job attraction.

Finally, the results of our study could inform how jobs are designed and advertised, to increase the likelihood that younger and older people apply for open job positions in the first place. In addition, our results may provide advice to companies on how to (re-)structure jobs so that younger and older workers are more likely to stay with the organization, are more satisfied with their job conditions, and potentially work more efficiently (Zacher and Schmitt, 2016).

## Job characteristics and age

Organizational researchers have argued that job characteristics influence employees' psychological states and, in turn, work outcomes such as job satisfaction, strain, absenteeism, and turnover (Hackman and Oldham, 1976; Fried and Ferris, 1987; Steyn and Vawda, 2014). Chapman et al. (2005) showed in their meta-analysis that job attraction is influenced directly by job and position characteristics (i.e., compensation and advancement, pay, type of work) and organizational characteristics (i.e., work environment, organizational image, location, size, familiarity, work hours). However, these researchers did not include motivational job characteristics in their meta-analysis.

Morgeson and Humphrey (2006) reviewed an extensive array of important job characteristics and combined several characteristics in a comprehensive measurement tool, the Work Design Questionnaire (WDQ). In our study, we focus on four job characteristics included in both job characteristics theory (Hackman and Oldham, 1976) and the WDQ, which have been identified as having age-differential effects on work outcomes by Truxillo et al. (2012). Specifically, we included four job characteristics (i.e., job autonomy, task variety, task significance, feedback from the job) that belong to the broader category of task characteristics. According to Morgeson and Humphrey (2006), task characteristics include those features of the job that describe how the work itself is done and the nature and breadth of tasks in a job (in addition to task characteristics, Morgeson and Humphrey (2006) include knowledge characteristics, social characteristics, and contextual characteristics).

### Job autonomy

Job autonomy refers to the extent to which workers are able to independently make decisions, and have autonomy in planning and carrying out their work tasks (Hackman and Oldham, 1976; Morgeson and Humphrey, 2006). Job autonomy has been shown to be positively related to job satisfaction and work motivation (Humphrey et al., 2007). In their model, Truxillo et al. (2012) suggest that job autonomy has a stronger positive effect on job satisfaction and performance among older compared to younger workers. Older workers typically have been working in their jobs for longer and therefore are more interested in autonomy to make use of their experiential knowledge and skills (see also Zacher and Frese, 2009). They further proposed that younger workers are still gaining work experience and have a higher need for supervision and thus expect less autonomy. Furthermore, Truxillo et al. (2012) suggested that older workers value job autonomy more than younger workers because it allows them to adapt to job demands and possibly compensate for age-related limitations, such as decreases in physical strength and fast information processing abilities (Kanfer and Ackerman, 2004). Therefore, job autonomy should be more attractive to older compared to younger workers. Consistent with these assumptions, a study by Zaniboni et al. (2016) showed that job autonomy was stronger positively related to the job satisfaction of older compared to younger construction workers. Job satisfaction, in turn, was positively related to mental health.

*Hypothesis 1*: *The positive effect of job autonomy on job attraction is moderated by age, such that the effect is stronger for older compared to younger workers*.

### Task variety

Task variety describes the diversity of the job requirements, that is, how many different tasks a worker is expected to perform as part of the job (Hackman and Oldham, 1976; Morgeson and Humphrey, 2006). Jobs that have higher levels of task variety are generally assumed to be more pleasant to perform (Humphrey et al., 2007). Lifespan theories (e.g., Carstensen et al., 1999) propose that younger workers will find high task variety more useful than older workers, as they have yet to gain experience in different tasks, whereas older workers already have acquired skills necessary for the job (see also Truxillo et al., 2012). Thus, older workers might see task variety as a burden in that they have to fulfill tasks that do not focus on their existing experience and specialized expertise. Task variety should therefore be more attractive for younger compared to older workers. This proposition has been supported by survey research which found that task variety has a stronger influence on younger workers' job satisfaction (Zaniboni et al., 2013, 2014).

*Hypothesis 2*: *The positive effect of task variety on job attraction is moderated by age, such that the effect is stronger for younger compared to older workers*.

### Task significance

Task significance refers to the influence and impact that people's jobs have on other people's lives or work (Hackman and Oldham, 1976; Morgeson and Humphrey, 2006). Perceptions of task significance are thought to enhance workers' experience of meaningfulness, which is believed to mediate the relationship between task significance and work outcomes (Humphrey et al., 2007). Furthermore, task significance is positively related to job satisfaction, work motivation, and performance (Humphrey et al., 2007; Grant, 2008). Based on the lifespan theory of socioemotional selectivity (Carstensen et al., 1999), Truxillo et al. (2012) argued that older workers are more likely to value task significance in a job than younger workers. Workers are increasingly looking for meaning in their jobs as they get older (and their future time perspective becomes more limited), whereas younger workers (who typically have higher levels of future time perspective) are more focused on acquiring new and useful skills and various job-related experiences.

*Hypothesis 3*: *The positive effect of task significance on job attraction is moderated by age, such that the effect is stronger for older compared to younger workers*.

### Feedback from the job

Feedback from the job reflects the extent to which workers receive direct and explicit feedback on how effectively they are performing the required tasks (Hackman and Oldham, 1976; Morgeson and Humphrey, 2006). Feedback from the job refers to feedback that is obtained through the results of a worker's performance rather than feedback given by other people (Morgeson and Humphrey, 2006). Feedback has a positive influence on job satisfaction, work motivation, as well as job performance (Humphrey et al., 2007). Truxillo et al. (2012) suggested in their model that feedback from the job will be particularly valued by younger workers as they still lack work experience and seek feedback to improve their performance to further their careers. In contrast, older workers are more experienced and have more confidence regarding their performance and therefore need less feedback (Wang et al., 2015). Feedback should therefore be more attractive for younger compared to older workers.

*Hypothesis 4*: *The positive effect of feedback from the job on job attraction is moderated by age, such that the effect is stronger for younger compared to older employees*.

## Methods

We used a policy-capturing design, a specific design that is part of the broader category of experimental vignette methodology designs, to test our hypotheses. Experimental vignette methodology designs can be used to assess behaviors, attitudes, and intentions in experimental settings while improving experimental realism through the construction of realistic scenarios (Aguinis and Bradley, 2014). Aguinis and Bradley (2014) suggest that policy-capturing designs are a particularly useful method for assessing implicit decision-making processes.

In the present study, we created hypothetical scenarios in which each of the four job characteristics (i.e., job autonomy, task variety, task significance, and feedback from the job) was manipulated (Karren and Barringer, 2002). Specifically, a number of scenarios was created using statements from the German version of the WDQ (Morgeson and Humphrey, 2006; Stegmann et al., 2010). For each scenario, participants were asked to rate the attractiveness of the job described in the scenario. In line with best practices (Aiman-Smith et al., 2002; Rotundo and Sackett, 2002; Ohme and Zacher, 2015), we conducted a pilot study before the main study to validate the statements used in the scenarios.

Both the pilot study and the main study were reviewed and approved by the Ethical Committee Psychology at the University of Groningen (Netherlands; see http://www.rug.nl/research/heymans-institute/organization/ecp/?lang$=$en). All participants gave written informed consent in accordance with the Declaration of Helsinki.

### Pilot study

#### Participants and procedure

In total, 20 participants completed the pilot study after they were provided with a link to an online survey. Participants were recruited through personal and professional contacts in Germany. No demographic data were collected.

#### Materials and measures

Statements describing different levels of each of the four job characteristic were shown to the pilot study participants. For each job characteristic, three statements were chosen randomly from the respective items provided by the WDQ (Morgeson and Humphrey, 2006). Each statement was shown with three different levels of intensity (low, medium, high). Thus, there were nine statements per job characteristic. Participants were asked, “How much autonomy does this job offer?,” “How much task variety does this job offer?,” “How significant or important is this job?,” and “How much feedback does this job offer?,” respectively. Participants were asked to indicate their answers on 7-point scales ranging from “very little” (1) to “very much” (7). Table 1 shows the wording of the items and descriptive statistics of the pilot study.

**Table 1:** Descriptive statistics from pilot study (*N* = 20).

**Job characteristics/Items**	**Low level**		**Medium level**		**High level**		**Difference low-medium**	**Difference medium-high**
	***M***	***SD***	***M***	***SD***	***M***	***SD***	***t***	***t***
**JOB AUTONOMY**								
The job (very rarely/sometimes/very often) gives me a chance to use my personal initiative or judgment in carrying out the work.	1.53	0.77	3.68	0.75	6.16	0.77	−12.30^***^	−13.96^***^
The job allows me to make (very few/some/a lot of) decisions on my own.	1.37	0.76	3.74	0.65	6.63	0.50	−12.43^***^	−22.25^***^
The job provides me with (very little/moderate/significant) autonomy in making decisions.	1.74	1.28	4.11	0.46	6.32	1.16	−8.52^***^	−7.84^***^
**TASK VARIETY**								
The job involves performing a (low/moderate/great) variety of tasks.	1.30	0.47	4.15	0.49	6.45	0.76	−26.05^***^	−14.04^***^
The job (very rarely/sometimes/very often) involves doing a number of different things.	1.25	0.44	4.00	0.80	6.55	0.61	−15.64^***^	−12.07^***^
The job (very rarely/sometimes/very often) requires the performance of a wide range of tasks.	1.30	0.57	4.00	0.46	6.35	0.75	−16.48^***^	−15.67^***^
**TASK SIGNIFICANCE**								
The results of my work (very rarely/sometimes/very often) significantly affect the lives of other people.	2.35	1.27	4.20	0.41	6.35	0.81	−6.75^***^	−10.30^***^
The work performed on the job has a (very small/moderate/significant) impact on people outside the organization.	2.40	1.43	4.35	0.75	6.25	0.79	−7.61^***^	−7.03^***^
The job has a (little/moderate/large) impact on people outside the organization.	2.37	1.17	4.11	0.81	6.47	0.84	−5.90^***^	−10.21^***^
**FEEDBACK FROM THE JOB**								
The work activities themselves provide (very rarely/sometimes/very often) direct and clear information about the effectiveness (e.g., quality and quantity) of my job performance.	1.50	0.76	3.95	0.69	6.45	0.76	−10.97^***^	−9.38^***^
The job itself (very rarely/sometimes/very often) provides feedback on my performance.	2.45	1.57	3.90	0.64	6.20	1.06	−3.81^***^	−9.52^***^
The job itself provides me (very rarely/sometimes/very often) with information about my performance.	1.90	0.91	3.90	0.85	6.55	0.76	−7.65^***^	−11.40^***^

Items were adapted from the Work Design Questionnaire (Morgeson and Humphrey, 2006). Items were followed by the questions: “How much autonomy does this job offer?,” “How much task variety does this job offer?,” “How significant is this job?,” and “How much feedback does this job offer?,” respectively. Responses were provided on 7-point scales ranging from 1 (very little) to 7 (very much).

*** *p < 0.001*.

Results of a series of *t*-tests showed that the differences between participants' ratings of low intensity statements vs. medium intensity statements, as well as the differences between participants' rating of medium intensity statements vs. high intensity statements for each of the job characteristic statements were significant and consistent with expectations, in that low intensity statements were rated lower than medium intensity statements and medium intensity statements were rated lower than high intensity statements (see Table 1). Thus, we concluded that it was acceptable to use the statements to create the scenarios for our main study.

### Main study

#### Participants and procedure

We recruited a convenience sample for the main study, again relying on personal and professional contacts in Germany. Participants were contacted by the second author and asked whether they would be willing to take part in a research study on job design. After a short introduction and explanation of the study, participants were provided with a link to an online survey. In total, 114 people started the online survey and answered at least one question. However, only 82 workers provided sufficient information on the study variables to be included into the analyses. All participants were employed or self-employed. There were 46 female participants and 36 male participants. Ages ranged from 19 to 65 years (*M* = 41.41, *SD* = 14.08). Participants had been working in their current jobs for an average of 14.44 years (*SD* = 12.57). The sample was highly educated with 42 of the participants (51.2%) holding a university degree, and another 20 (24.4%) of the participants having acquired the German general qualification for entering university. Participants' professions were very diverse. For example, job descriptions included teachers, lawyers, administrators, and biologists.

#### Materials and measures

**Figure 1:**
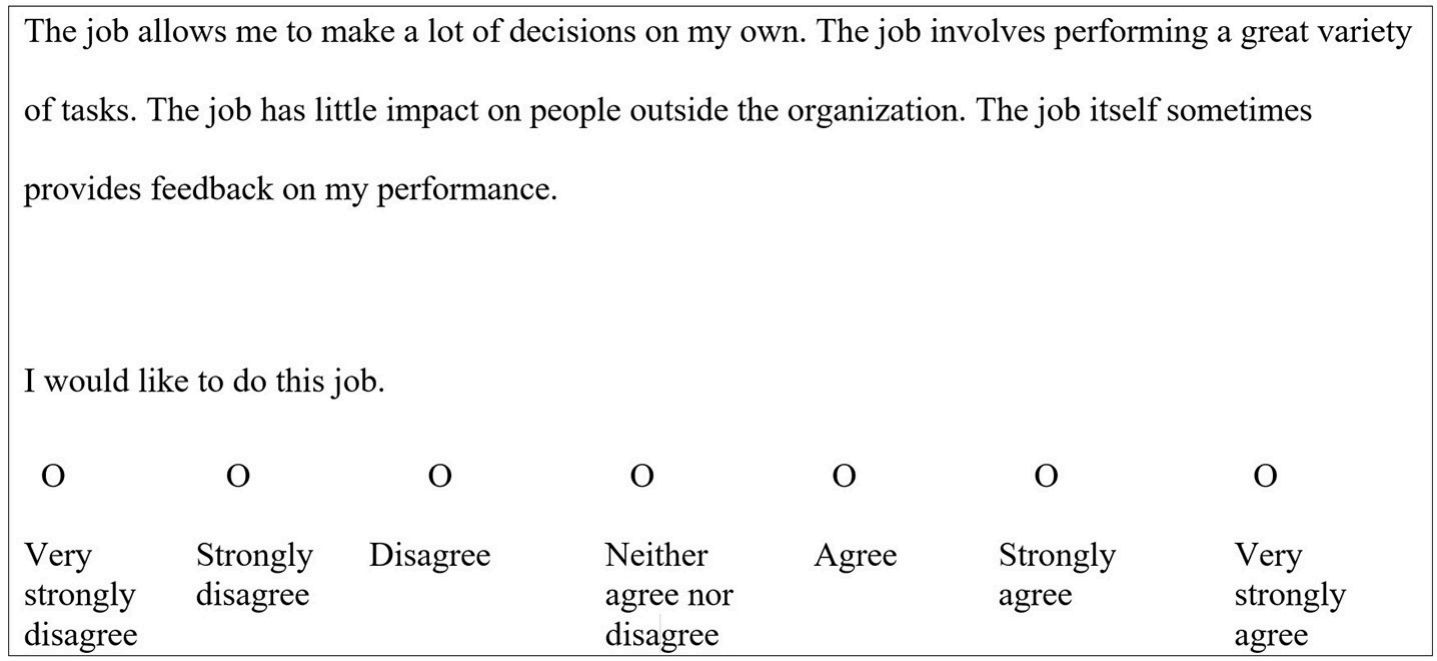
Example Scenario. The scenario describes a job with high levels of job autonomy and task variety, as well as low levels of task significance and medium levels of feedback from the job.

We set up an online survey in which participants were first asked to answer some general demographic questions, including age, gender, education, job tenure, and job description. Subsequently, participants were instructed to read a series of descriptions of hypothetical job descriptions and rate how much they would like to carry out these jobs. Each job description included four statements validated in the pilot study; the statements presented different levels of job autonomy, task variety, task significance, and feedback from the job. As in the pilot study, for each job characteristic there were three different statements based on the WDQ items (Morgeson and Humphrey, 2006), and for each statement there were three different levels of intensity: low, medium, and high. The statements for each scenario were chosen randomly with regard to the statement itself, as well as with regard to the intensity level of each job characteristic. In total, we created 41 scenarios, including one duplicate scenario to assess reliability (Rotundo and Sackett, 2002; Ohme and Zacher, 2015). Each scenario contained four randomly selected and randomly ordered statements. This was done to avoid possible primacy or recency effects (Rotundo and Sackett, 2002). The order in which the scenarios were presented to participants was also randomized. Participants provided their ratings of job attraction on a 7-point Likert scale ranging from “very strongly disagree” (1) to “very strongly agree” (7). An example scenario is shown in Figure 1. Overall, 82 participants provided 3,280 ratings, suggesting that all participants rated all 40 job descriptions.

As participants' demographic characteristics and characteristics of their current jobs may influence job attraction (Chapman et al., 2005), we controlled in the analyses for gender (1 = *male*, 2 = *female*), education (ranging from 1 = *no school degree* to 5 = *university degree*), job tenure (in years), and the characteristics of participants current jobs. We coded job descriptions provided by participants using the Occupational Information Network (O^*^Net) database (Peterson et al., 2001; see https://www.onetonline.org/) and a coding scheme developed by Gonzalez-Mulé (2015). Specifically, as outlined by Gonzalez-Mulé (2015), we used values of items from the work activities and work context inventories of O^*^NET that correspond to the four job characteristics of interest. Specifically, job autonomy was measured with the item “freedom to make decisions” (i.e., how much decision making freedom, without supervision, does the job offer?); task variety was measured with the item “importance of repeating the same task” (reverse coded; i.e., how important is repeating the same physical activities…or mental activities…over and over, without stopping, to performing this job?); task significance was measured with the item “impact of decisions on co-workers or company results” (i.e., what results do your decisions usually have on other people or the image or reputation or financial resources of your employer?); and feedback from the job was measured with the item “making decisions and solving problems” (i.e., how often workers receive feedback on their performance and act upon it; see Table 5 in Gonzalez-Mulé, 2015). The values of the O^*^Net items were obtained from a large random sample of job incumbents who rated the extent to which their jobs are characterized by the different descriptors. Previous research has demonstrated the high reliability of the O^*^Net inventories (Childs et al., 1999; Strong et al., 1999; Peterson et al., 2001).

#### Statistical analyses

Data were analyzed with random coefficient (i.e., multilevel) models using the hierarchical linear modeling software (Hofmann et al., 2000; Raudenbush et al., 2011), because scenario ratings were nested within participants and the software supports the analysis of both within- as well as between-person variance (Kristof-Brown et al., 2002; Rotundo and Sackett, 2002). The within-person predictors of job attraction (i.e., the independent variables of job autonomy, task variety, task significance, and feedback from the job) were group-mean centered. Age as a between-person predictor and moderator variable was centered at the grand mean (the control variables were also grand mean centered). To probe significant interaction effects, we created plots of the regions of significance, which show the simple slopes (i.e., effect of job characteristic on job attraction) for different values of the moderator variable (i.e., age; Bauer and Curran, 2005; Preacher et al., 2006). In addition, this plotting technique may help detect potential curvilinear moderating effects of age (Rauschenbach et al., 2013; Zacher and Schmitt, 2016).

## Results

### Preliminary analyses

Descriptive statistics and correlations of the study variables are shown in Table 2. Of note, at the bivariate and between-person level, aggregated job attraction ratings were negatively related to age, job tenure, and job autonomy (O^*^Net). Thus, older workers, as well as workers with higher job tenure and higher job autonomy generally rated the hypothetical job descriptions less favorably.

**Table 2:** Descriptive statistics and correlations.

**Variable**	***M***	***SD***	**1**	**2**	**3**	**4**	**5**	**6**	**7**	**8**	**9**
1. Job attraction	3.57	0.58	–								
2. Age	41.41	14.08	−0.37^**^	–							
3. Gender^a^	1.56	0.50	0.16	−0.34^**^	–						
4. Education	4.24	0.88	0.03	−0.07	0.19	–					
5. Job tenure	14.44	12.57	−0.28^*^	0.82^**^	−0.30^**^	−0.34^**^	–				
6. Job autonomy (O^*^Net)	81.49	9.79	−0.25^*^	0.19	0.10	0.08	0.06	–			
7. Task variety (O^*^Net)	40.65	16.75	−0.10	0.08	−0.05	0.26^*^	−0.07	0.14	–		
8. Task significance (O^*^Net)	71.84	12.86	−0.10	0.01	0.11	0.14	−0.09	0.70^**^	0.26^*^	–	
9. Feedback from the job (O^*^Net)	63.17	11.40	−0.09	0.07	−0.18	0.11	−0.03	0.30^**^	0.38^**^	0.59^**^	–

N = 82. ^a^1, male; 2, female. O^*^Net, Occupational Information Network (https://www.onetonline.org).

** p < 0.01;

* *p < 0.05*.

We first ran a null (or intercept-only) model to test whether the use of multilevel modeling was appropriate. The chi-square test for the intercept (*r*_0_) was significant, $\chi_{\left(81\right)}^{2}$ = 473.90 with *p* < 0.001, and the intraclass correlation coefficient was 0.12 (see Table 3). This value indicates that approximately 12% of the variance in job attraction can potentially be explained by between-person factors (e.g., participants' age), leaving approximately 88% of the variance that could potentially be explained by within-person factors (i.e., the job characteristics in our study). Thus, the use of multilevel modeling in our study was appropriate.

**Table 3:** Results of multilevel analysis predicting job attraction.

**Predictor**	**Null model**			**Predictor model**		
	**γ**	***SE***	***t***	**γ**	***SE***	***t***
Intercept	3.55	0.06	56.36^***^	3.55	0.06	59.54^***^
**BETWEEN-PERSON PREDICTOR AND CONTROL VARIABLES**						
Age				−0.01	0.01	−1.39
Gender				0.09	0.14	0.63
Education				0.00	0.08	0.04
Job tenure				−0.00	0.01	−0.03
Job autonomy (O*Net)				−0.01	0.01	−1.65
Task variety (O*Net)				−0.00	0.00	−0.54
Task significance (O*Net)				0.00	0.01	0.52
Feedback from the job (O*Net)				−0.00	0.01	−0.13
**WITHIN-PERSON PREDICTORS**						
Job autonomy				0.73	0.03	27.74^***^
Task variety				0.58	0.03	19.87^***^
Task significance				0.36	0.03	13.64^***^
Feedback from the job				0.37	0.03	14.47^***^
**CROSS-LEVEL INTERACTIONS**						
Job autonomy × Age				−0.00	0.00	−1.89
Task variety × Age				−0.01	0.00	−3.69^***^
Task significance × Age				−0.01	0.00	−4.99^***^
Feedback from the job × Age				−0.01	0.00	−3.12^***^
**VARIANCE COMPONENTS**						
Level 1 (σ^2^)		1.95			1.30	
Level 2 Intercept (τ_00_)		0.26			0.25	
**ADDITIONAL INFORMATION**						
ICC		0.12				
Pseudo *R^2^*					0.30	

N = 82 participants provided 3,280 job attraction. Unstandardized coefficients (γ) and standard errors (SE) are shown. ICC, intraclass correlation coefficient. The ICC is calculated by dividing the between-person variance component (τ_00_) of the null model (i.e., the model with no predictors) by the sum of τ_00_ and the within-person variance component (σ^2^) of the null model. Pseudo R^2^, proportion of variance explained in dependent variable by predictors at the between-person and within-person levels.

*** *p < 0.001*.

To assess test-retest reliability, we included a duplicate scenario in our study (see also Rotundo and Sackett, 2002; Ohme and Zacher, 2015). The duplicate scenario was not included in subsequent analyses. Results showed that Cronbach's alpha was α = 0.65, indicating acceptable test-retest reliability of the job attraction ratings.

### Main effects of age and job characteristics

Results of the multilevel analysis showed that, at the between-person level, neither age nor the other demographic characteristics and control variables significantly predicted job attraction (see Table 3). In contrast, at the within-person level, all four job characteristics positively predicted job attraction (Table 3). Specifically, we found positive and significant main effects of job autonomy (β_1_ = 0.73, *p* < 0.001), task variety (β_2_ = 0.58, *p* < 0.001), task significance (β_3_ = 0.36, *p* < 0.001), and feedback from the job (β_4_ = 0.37, *p* < 0.001). These findings suggest that job autonomy and task variety were somewhat more important predictors of job attraction than task significance and feedback from the job.

### Moderating role of age

**Figure 2:**
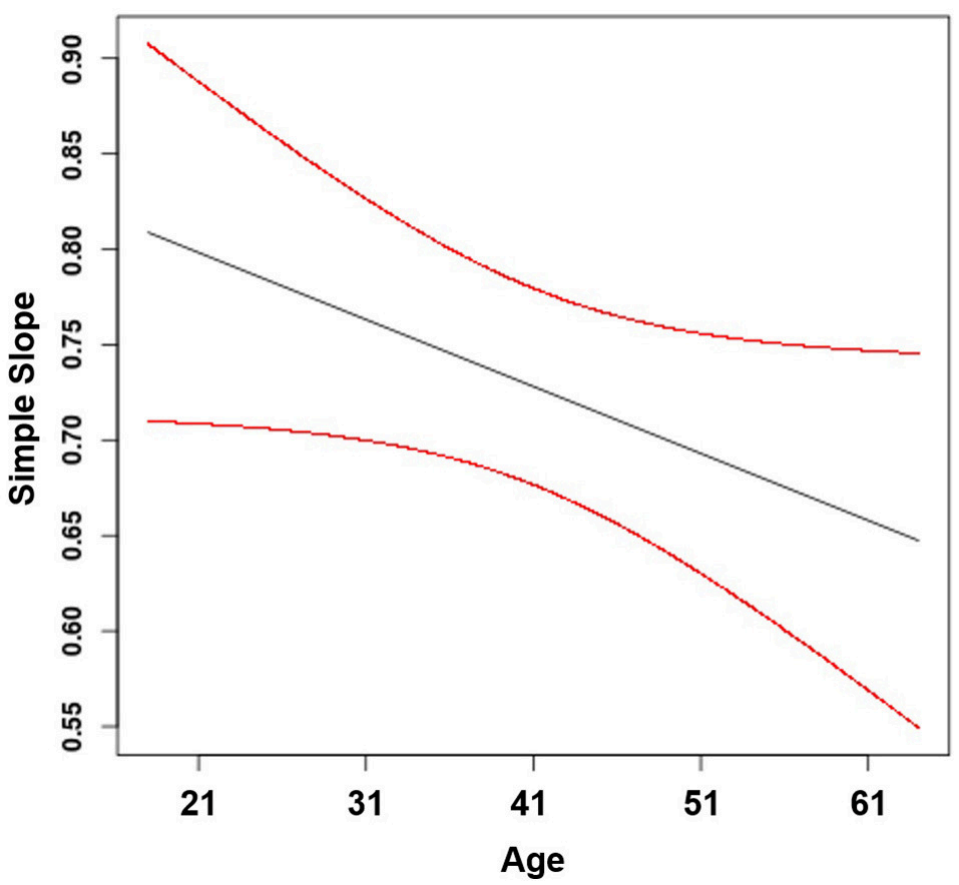
Effect of job autonomy on job attraction moderated by age (with 95% confidence bands).

Hypothesis 1 states that age moderates the positive effect of job autonomy on job attraction, such that the effect is stronger for older compared to younger workers. Results showed that the moderating effect of age was not significant (β_11_ = −0.00, *p* = 0.060; see Table 3). Thus, Hypothesis 1 did not receive support. Nevertheless, we plotted the effect of job autonomy on job attraction for different values of age, including younger workers (i.e., −1*SD* of age), middle-aged workers (i.e., mean age), and older workers (i.e., +1*SD* of age). Figure 2 shows that the simple slope was positive and significant for younger (γ = 0.78, *SE* = 0.04, *t* = 20.88, *p* < 0.001), middle-aged (γ = 0.73, *SE* = 0.03, *t* = 27.74, *p* < 0.001), and older workers (γ = 0.68, *SE* = 0.04, *t* = 18.37, *p* < 0.001). The non-significant interaction effect and the plot of the regions of significance suggest that the simple slopes did not differ for the various age groups included in our sample.

**Figure 3:**
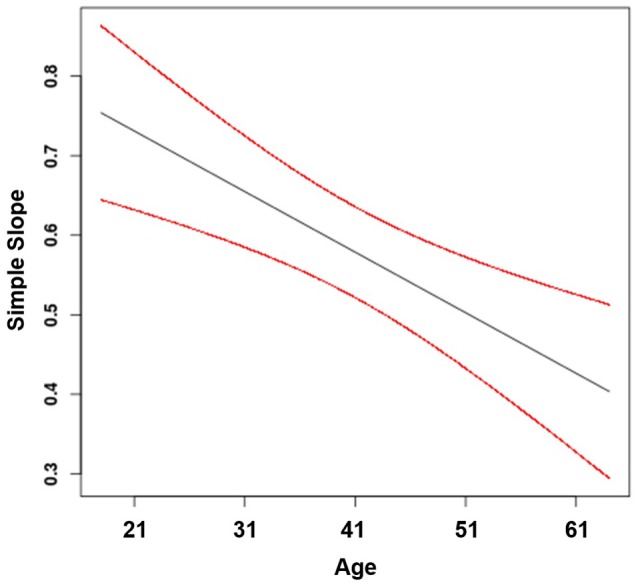
Effect of task variety on job attraction moderated by age (with 95% confidence bands).

According to Hypothesis 2, age moderates the positive effect of task variety on job attraction, such that the effect is stronger for young compared to older workers. Consistent with this hypothesis, we found a moderating effect of age (β_22_ = −0.01, *p* < 0.001; Table 3). As can be seen in Figure 3, the positive effect of task variety on job attraction was stronger for younger workers (γ = 0.69, *SE* = 0.04, *t* = 16.66, *p* < 0.001) than for middle-aged workers (γ = 0.58, *SE* = 0.03, *t* = 19.87, *p* < 0.001) and for older workers (γ = 0.47, *SE* = 0.04, *t* = 11.47, *p* < 0.001). In addition, the plot of the regions of significance suggests that the simple slopes were significant across all age groups included in our sample. Hypothesis 2 was, therefore, supported.

**Figure 4:**
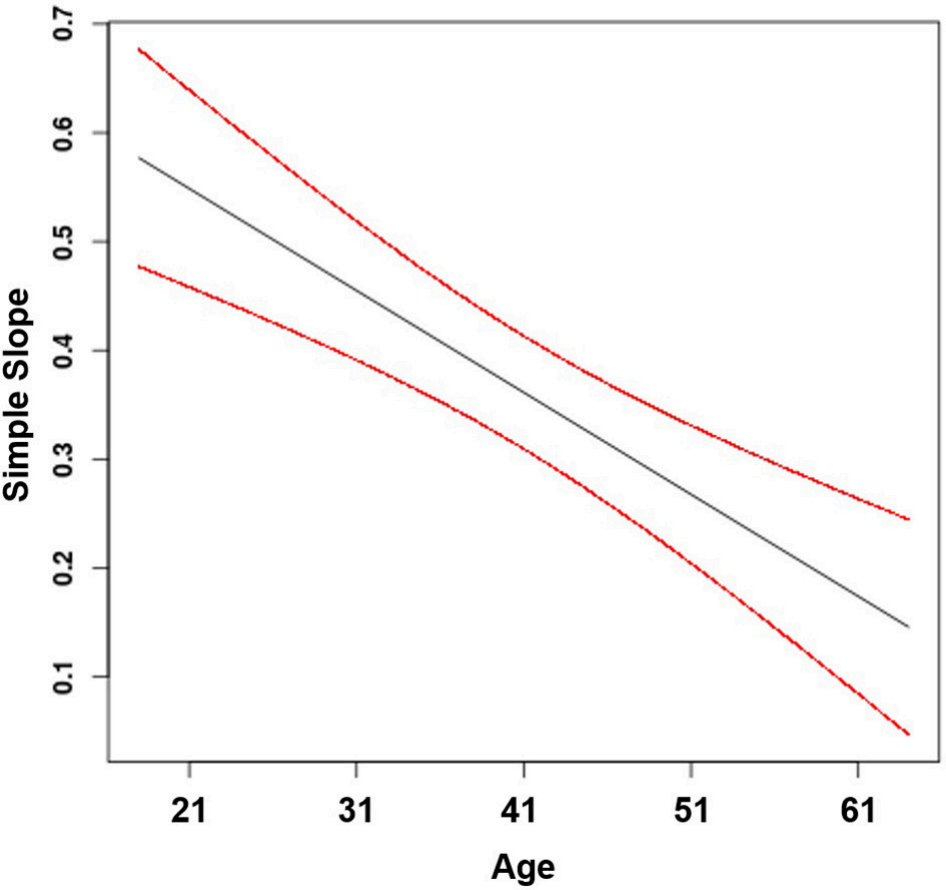
Effect of task significance on job attraction moderated by age (with 95% confidence bands).

Hypothesis 3 states that age moderates the positive effect of task significance on job attraction, such that the effect is stronger for older compared to younger workers. We found a significant moderating effect of age (β_33_ = −0.01, *p* < 0.001; Table 3). However, the positive effect of task significance on job attraction was stronger for younger workers (γ = 0.49, *SE* = 0.04, *t* = 59.54, *p* < 0.001) than for middle-aged workers (γ = 0.36, *SE* = 0.03, *t* = 13.64, *p* < 0.001) and for older workers (γ = 0.23, *SE* = 0.04, *t* = 6.16, *p* < 0.001; see Figure 4). Again, the plot of the regions of significance suggests that the simple slopes were significant across all age groups included in our sample. Thus, as we found the opposite to what we proposed, Hypothesis 3 was not supported.

**Figure 5:**
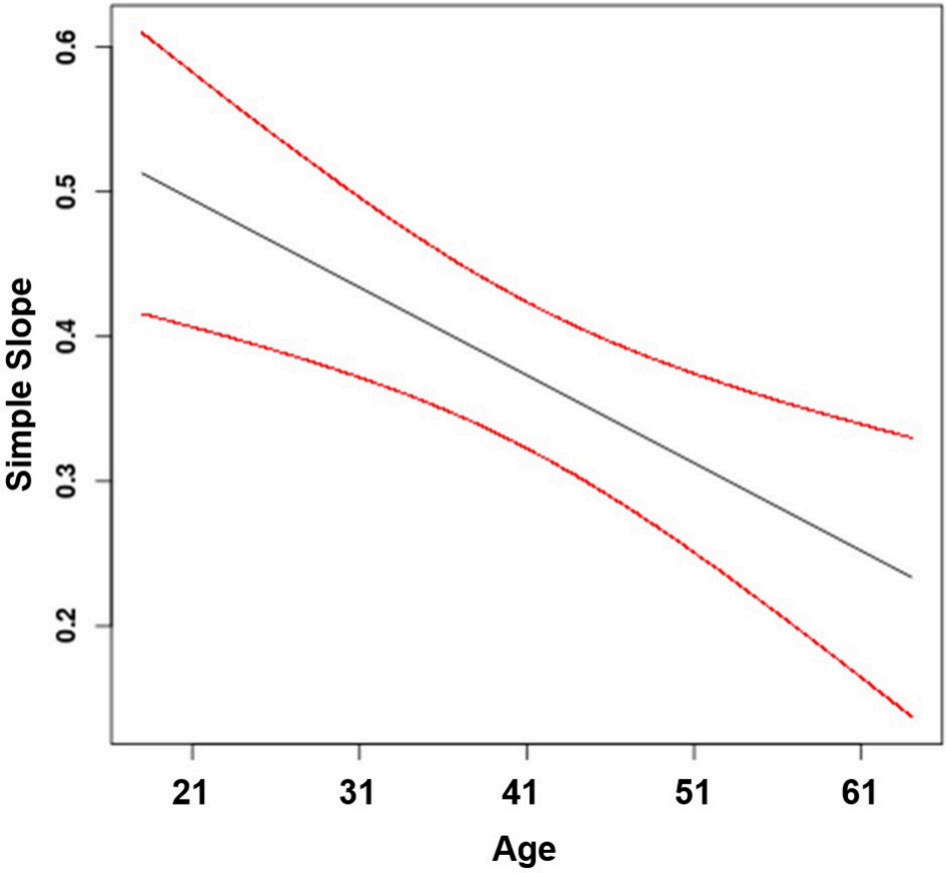
Effect of feedback from the job on job attraction moderated by age (with 95% confidence bands).

Finally, Hypothesis 4 proposes that age moderates the positive effect of feedback from the job on job attraction, such that the effect is stronger for younger compared to older workers. This hypothesis was supported by a significant moderating effect of age (β_44_ = −0.01, *p* < 0.001) and an interaction consistent with the hypothesized pattern. The plot of the regions of significance suggests that the simple slopes were significant across all age groups included in our sample (Figure 5). Specifically, the positive effect of feedback from the job on job attraction was stronger for younger workers (γ = 0.46, *SE* = 0.04, *t* = 12.55, *p* < 0.001) than for middle-aged workers (γ = 0.37, *SE* = 0.03, *t* = 14.47, *p* < 0.001) and for older workers (γ = 0.29, *SE* = 0.04, *t* = 7.93, *p* < 0.001).

Overall, according to the pseudo *R*^2^ statistic (LaHuis et al., 2014), age, control variables, job characteristics, and interactions between age and job characteristics explained 30% of the variance in job attraction (see Table 3).

## Discussion

### Summary and interpretation of findings

The goal of this study was to test core propositions of Truxillo et al. (2012) lifespan perspective on job design using a policy-capturing design, which is one type of experimental vignette methodology designs. Consistent with previous research on the positive effects of job characteristics on other work outcomes such as job satisfaction, work engagement, and job performance (Fried and Ferris, 1987; Humphrey et al., 2007), we found that job autonomy, task variety, task significance, and feedback from the job had positive main effects on participants' ratings of job attraction. Furthermore, we found age-differential effects for three out of the four job characteristics, of which two were consistent with the hypothesized pattern (i.e., those including task variety and feedback).

We did not find support for our first hypothesis, which stated that job autonomy has a stronger positive effect on job attraction among older compared to younger workers. It is important to note here that the interaction effect was not significant according to conventional cut-offs (*p* = 0.06) and, thus, statistical power may have been a problem. However, the interaction plot suggested that job autonomy has somewhat stronger effects among younger compared to older workers, which is contrary to our assumption based on the lifespan perspective on job design. In their model, Truxillo et al. (2012) suggested that older workers value job autonomy more than younger workers because they are more experienced and need less supervision compared to younger, less experienced workers. Consistently, Zaniboni et al. (2016) found that job autonomy was more important for older construction workers in terms of job satisfaction and mental health. However, meta-analytic research on age-differential effects of job autonomy on work outcomes has yielded mixed results (Ng and Feldman, 2015). For instance, the association between job autonomy and job performance was stronger for older workers, whereas the associations of job autonomy with job satisfaction and affective commitment were weaker for older workers. A potential explanation offered by Ng and Feldman (2015) for these mixed results is that the effects depend on the particular outcomes under study. It may be possible that job autonomy is particularly important for younger workers with regard to job attraction and other attitudinal outcomes, because most younger workers apply for a career job for the first time in their lives and, thus, motivational job characteristics such as autonomy may be more important to them than materialistic factors. In other words, it could be that younger people pay more attention to what makes a new job interesting, challenging, and important, as compared to factors such as pay and the physical work environment. In contrast, job autonomy may be more important for older workers with regard to job performance, because it allows them to make use of their accumulated knowledge, experience, and skills.

Second, we hypothesized that the positive effect of task variety on job attraction are stronger for younger compared to older workers. This hypothesis was supported, providing further support for the notion that task variety is more important for younger than older workers. For instance, survey research showed that younger workers with higher task variety are more satisfied with their jobs (Zaniboni et al., 2013, 2014); we extend this research by showing that younger workers are more attracted to jobs that promise to provide them with high levels of task variety. High task variety is particularly important for younger workers, because it allows them to gain diverse work-related experiences and develop new and useful skills (Truxillo et al., 2012).

Third, we expected that the effect of task significance on job attraction is stronger for older compared to younger workers. Our findings did not support this hypothesis, but instead showed the opposite pattern: younger workers were more attracted to jobs with higher task significance than older workers. This finding contradicts assumptions based on the lifespan theory of socioemotional selectivity (Carstensen et al., 1999). Specifically, Truxillo et al. (2012) argued that older workers value task significance more than younger workers as their limited future time perspective renders meaningfulness and intrinsic rewards more important than other job-related factors (e.g., pay). However, our findings are consistent with some recent research that suggested that younger workers are more interested in what influence their work has on other people and outside of the company (Scroggins, 2008; Murray et al., 2011). As noted above, it may also be the case that the motivational job characteristics we studied are particularly relevant for younger workers in terms of job attraction, because they focus more on motivational job characteristics (i.e., interesting, important, and challenging work) when evaluation a new career job as compared to materialistic factors (e.g., pay, physical work environment).

Finally, our hypothesis on age-differential effects of feedback from the job on job attraction was supported, suggesting that feedback from the job is more important for job attraction of younger compared to older applicants. This supports Truxillo et al.'s (2012) assumption that younger workers are less experienced and are interested in more feedback to improve their performance, whereas older workers already have a great amount of experience to rely on and hence need less feedback.

Taken together, we could confirm only two out of four hypotheses based on Truxillo et al.'s (2012) lifespan perspective on job design. While job autonomy did not have an age-differential effect on job attractiveness ratings, task variety, feedback, and, unexpectedly, task significance had stronger effects among younger compared to older workers. We extended research based on Truxillo et al.'s (2012) model, and the literature on recruitment more broadly, by focusing on job attraction as an outcome of motivational job characteristics. Moreover, the use of the policy-capturing method has advantages over survey designs, as it maximizes internal validity and realism (Aguinis and Bradley, 2014). Despite these strengths, our findings need to be interpreted in light of a number of limitations.

### Limitations and future research

First, participants had to read a large number of job descriptions. The hypothetical nature of these descriptions and increasing fatigue while participating in policy-capturing studies have frequently been mentioned as limitations of these designs (Kristof-Brown et al., 2002; Rotundo and Sackett, 2002). For example, Graham and Cable (2001) found that participants experienced more stress and felt more exhausted when responding to 32 scenarios as compared to only 8 scenarios. We took a number of precautions while designing the study to prevent fatigue from influencing our results, including relatively short descriptions and randomization of scenarios.

Second, critics may question the external validity and generalizability of studies using a policy-capturing design (Karren and Barringer, 2002). We asked participants to form a judgment about a hypothetical job description based on only four variables, whereas in reality applicants and workers may have access to more information about job openings. Furthermore, it could be argued that our participants, who were all currently working as employees or self-employed, may have answered differently if they were actually searching for a job at the time of answering the survey. Future research could replicate our study with jobseekers, possibly with realistic job advertisements. Moreover, future research could assess different or additional outcome variables, such as intentions to apply for a job opening (Chapman et al., 2005).

Third, we used a single item to assess job attraction, which may raise concerns about reliability. Some researchers have suggested that homogeneous constructs, such as global job satisfaction, can be reliably assessed using single items (Wanous et al., 1997; Fisher et al., 2016). We argue that job attraction is a rather homogeneous attitudinal construct that is distinct from other job-related attitudes and behavioral intentions (Highhouse et al., 2003; Chapman et al., 2005). Moreover, previous research has used similar single item measures to assess job attraction (Singh, 1975; Rynes and Lawler, 1983; Rynes and Miller, 1983). Also, due to the time-intensive nature of this approach, many policy-capturing studies use single items and report test-retest reliabilities across scenarios (Rotundo and Sackett, 2002; Ohme and Zacher, 2015). The test-retest reliability was acceptable in our study.

Fourth, consistent with Truxillo et al.'s (2012) lifespan model of job design, we did not include task identity (i.e., the extent to which workers perform complete tasks, including goal setting, planning, execution, and feedback processing; Hackman and Oldham, 1976; Hacker, 1986; Morgeson and Humphrey, 2006) in our study. Future research could focus on the importance of tasks identity among younger and older workers (see Zacher et al., 2016).

Finally, further research is needed that compares our current findings with age-differential effects of job characteristics with regard to different work outcomes, such as job satisfaction, work engagement, and job performance. The difference between these outcomes and job attraction is that the former outcomes can only be answered by job incumbents, whereas job attraction is mainly relevant among job seekers and job applicants who are not yet in a concrete work role. Moreover, researchers could investigate whether additional moderators of the job characteristics-outcome relationships may play a role. For instance, Truxillo et al. (2012) suggested that age-related factors such as future time perspective, self-regulatory strategies, and socioemotional selectivity processes (cf. Rudolph, 2016) may mediate the moderating effect of age. Furthermore, they proposed that additional individual differences (e.g., personality, health, cognitive abilities) and contextual factors (e.g., organizational culture, climate), as well as interactions among different job characteristics may act as boundary conditions of the age-differential effects of job characteristics on work outcomes.

### Practical implications and conclusion

Our study provides useful information for recruiters and human resource managers interested in addressing the challenges of an aging and increasingly age diverse workforce. Our findings suggest that younger workers value certain job characteristics more than older workers, including task variety and feedback from the job. Thus, organizations aiming to recruit younger job applicants could adjust their job advertisements by emphasizing high levels of task variety and feedback from the job (i.e., targeted recruitment; Newman and Lyon, 2009). At the same time, younger, middle-aged, and older applicants appear to value job autonomy to a similar extent. Employers could use this information in the recruitment process to gain more interest from applicants by advertising the position as providing high levels of job autonomy.

In summary, our findings suggest that younger and older workers differ with regard to their preferences for task variety, task significance, and feedback from the job, but not job autonomy. Specifically, younger workers rated jobs with high levels of task variety, task significance, and feedback from the job as more attractive than older workers. So far, most research on age and job characteristics has focused on how to design jobs to motivate older employees. In contrast, our findings provide practitioners with suggestions on how to change job characteristics to make jobs more attractive to younger job applicants, as well as workers of all ages.

## Author contributions

HZ and BD designed the study. BD collected the data. HZ analyzed the data and wrote the first draft of the manuscript, and SK and BH revised the manuscript.

### Conflict of interest statement

The authors declare that the research was conducted in the absence of any commercial or financial relationships that could be construed as a potential conflict of interest.
